# Anionic Complex with Efficient Expression and Good Safety Profile for mRNA Delivery

**DOI:** 10.3390/pharmaceutics13010126

**Published:** 2021-01-19

**Authors:** Eri Hamada, Tomoaki Kurosaki, Junya Hashizume, Hitomi Harasawa, Hiroo Nakagawa, Tadahiro Nakamura, Yukinobu Kodama, Hitoshi Sasaki

**Affiliations:** 1 Graduate School of Biomedical Sciences, Nagasaki University, 1-7-1 Sakamoto, Nagasaki 852-8588, Japan; bb55317021@ms.nagasaki-u.ac.jp (E.H.); kurosaki@nagasaki-u.ac.jp (T.K.); 2 Department of Hospital Pharmacy, Nagasaki University Hospital, 1-7-1 Sakamoto, Nagasaki 852-8501, Japan; hassy1984-ngs@umin.net (J.H.); harasawa-ngs@umin.ac.jp (H.H.); 07-07@umin.net (H.N.); t-nakamr@nagasaki-u.ac.jp (T.N.); y-kodama@nagasaki-u.ac.jp (Y.K.)

**Keywords:** mRNA, nanoparticles, polyethyleneimine, γ-polyglutamic acid

## Abstract

We previously found that a complex comprising plasmid DNA (pDNA), polyethylenimine (PEI), and γ-polyglutamic acid (γ-PGA) had high transgene efficiency without cytotoxicity in vitro and in vivo. However, messenger RNA (mRNA) remains an attractive alternative to pDNA. In this study, we developed a safe and effective delivery system for mRNA to prevent its degradation and efficiently deliver it into target cells. Various cationic and anionic complexes were produced containing PEI, γ-PGA, and an mRNA encoding firefly luciferase. Their physicochemical properties and cytotoxicities were analyzed and the in vitro and in vivo protein expression were determined. The cationic mRNA/PEI complex showed high in vitro protein expression with strong cytotoxicity. The anionic complex was constructed as mRNA/PEI8/γ-PGA12 complex with a theoretical charge ratio of 1:8:12 based on the phosphate groups of the mRNA, nitrogen groups of PEI, and carboxylate groups of γ-PGA. It was stable and showed high in vitro protein expression without cytotoxicity. After intravenous administration of mRNA/PEI8/γ-PGA12 complex to mice, high protein expression was observed in the spleen and liver and slight expression was observed in the lung over 24 h. Thus, the newly constructed mRNA/PEI8/γ-PGA12 complex provides a safe and effective strategy for the delivery of mRNA.

## 1. Introduction

Gene therapies are next-generation drugs that express targeted protein products for protein replacement therapy, vaccination, and the treatment of genetic diseases [1]. Messenger RNA (mRNA) is more suitable for clinical application than DNA [2]. Because mRNA cannot be integrated into the host genome, mRNA does not pose any danger of insertion mutations. Furthermore, mRNA only requires delivery into the cytosol of the host cells because transcription is not necessary for mRNA. However, mRNA is a water-soluble polymer, and it is rapidly degraded by nucleases found throughout tissues and organs [3]. Very poor cellular uptake of mRNA has been reported, and this has been attributed to its negative charge repelling the cell membrane [4,5]. Therefore, there remains scope to develop a more useful approach for targeted mRNA delivery that is both efficient and safe.

The chemical modification of mRNA is one such approach, although challenges remain because of off-target effects and low selectivity of the target cells in in vivo applications [6,7]. Another promising approach is to develop in vivo mRNA delivery systems comprising complexes of mRNA with cationic lipids and cationic polymers, although these have been shown to have strong cytotoxicity and hematotoxicity [8,9]. This can be alleviated by the addition of several anionic polymers to the cationic complexes, but this has been reported to reduce the interaction of these complexes with the anionic cellular membrane [10,11,12]. Thus, while most anionic complexes of mRNA do not show cytotoxicity, they exhibit markedly reduced protein expression due to poor cellular uptake.

Previously, we found that the addition of γ-polyglutamic acid (γ-PGA) to a cationic complex of plasmid DNA (pDNA) with polyethyleneimine (PEI) formed a special anionic complex that showed high protein expression without cytotoxicity, hematotoxicity, or hepatotoxicity [13,14]. Following intravenous injection of this anionic complex of pDNA, PEI, and γ-PGA into mice, the complex showed high accumulation and strong protein expression in the splenic marginal zone, which is the region at the interface between the non-lymphoid red pulp and the lymphoid white pulp.

This delivery system, which was specific to the spleen, was applied to DNA vaccination for melanoma [14]. Melanoma, a malignant neoplasm of melanocytes, is the most dangerous type of skin cancer and is highly metastatic and resistant to conventional therapy [15,16,17]. We used a pDNA (pUb-M) encoding melanoma-related antigen to construct the melanoma DNA vaccine. In the B16-F10 mouse model of metastatic melanoma, vaccination with this complex significantly inhibited lung metastasis. No acute toxicity or liver injury was observed following intravenous injections of the complex [14].

This splenic delivery system for pDNA was also applied to malaria vaccination [18]. We used a pDNA (pVAX-MSP-1) encoding *Plasmodium yoelii* merozoite surface protein-1. Following vaccination with this complex and lethal challenge with *Plasmodium yoelii*, 60% of the infected mice survived. The cytokines IL-12p40, IFN-γ, and IL-4 were induced in the serum of the vaccinated mice, and an antigen-specific IgG1 and IgG2 response was also observed.

In comparison with pDNA, mRNA is more prone to degradation and, as a result, low delivery into the target cells. The process of protein expression from mRNA is different from that of pDNA. Therefore, to develop an optimal anionic mRNA complex, we constructed various complexes containing PEI, γ-PGA, and an mRNA encoding firefly luciferase and investigated their physicochemical characteristics, nuclease tolerance, cytotoxicity, hematotoxicity, and in vivo protein expression in mice. The mRNA/PEI8/γ-PGA12 complex showed low cytotoxicity and high protein expression in vitro. In addition, after intravenous administration of the mRNA/PEI8/γ-PGA12 complex to mice, high protein expression was observed in the liver and spleen. This mRNA complex has potential for applications in liver enzyme replacement therapy and vaccine delivery.

## 2. Materials and Methods

### 2.1. Chemicals

The mRNA encoding firefly luciferase was purchased from TriLink BioTechnologies (San Diego, CA, USA). Branched PEI with a molecular weight of 25 kDa was provided by Aldrich Chemical Co. (Milwaukee, WI, USA). The γ-PGA (molecular weight, 10–100 kDa) was obtained from Yakult Pharmaceutical Industry Co., Ltd. (Tokyo, Japan). All other chemicals were of reagent grade.

### 2.2. Preparation of Complexes

We prepared various complexes at a theoretical charge ratio based on the phosphate groups of the mRNA, nitrogen groups of PEI, and carboxylate groups of γ-PGA. To construct the mRNA/PEI complexes, the mRNA solution (1 mg/mL in 1 mM sodium citrate buffer) and the PEI solution (1 mg/mL in 5% glucose solution) were mixed in a 5% glucose solution by thorough pipetting and this mixture was incubated for 30 min on ice to produce mRNA/PEI complexes with charge ratios of 1:2, 1:4, 1:6, 1:8, 1:10, or 1:12 (termed the mRNA/PEI2, 4, 6, 8, 10, or 12 complex, respectively). To prepare the mRNA/PEI/γ-PGA complexes, the γ-PGA solution (10 mg/mL in a 5% glucose solution) was added to the mRNA/PEI8 complex solution at a charge ratio of 1:8:2, 1:8:4, 1:8:6, 1:8:8, 1:8:10, or 1:8:12 (termed the mRNA/PEI8/γ-PGA2, 4, 6, 8, 10, or 12 complex, respectively), and this mixture was left for another 30 min on ice.

### 2.3. Physicochemical Properties of the Complexes

The particle size and ζ-potential of each complex were measured with a Zetasizer Nano ZS (Malvern Instruments, Ltd., Malvern, UK). The number-fractioned mean diameter is shown.

To assess complex formation, each complex solution containing 1 µg of mRNA was loaded onto a 1% agarose gel and electrophoresis was performed using the i-Mupid J system (Cosmo Bio, Tokyo, Japan). Retardation of the mRNA was visualized with GelRed (Biotium, Fremont, CA, USA) and the Gel Doc EZ Imager (Bio-RAD, Hercules, CA, USA). Degradation of the mRNA was determined by incubating the mRNA/PEI8/γ-PGA12 complex containing 1 μg of mRNA with 1 × 10^−7^ mg/mL RNase for 30 min at 37 °C. This mixture was then electrophoresed as described above. To release mRNA from the complex, 5 µg heparin sulfate was added.

### 2.4. In Vitro Protein Expression

The B16 mouse melanoma cell line was provided by the Cell Resource Center for Biomedical Research (Tohoku University, Miyagi, Japan). The cells were cultured in RPMI 1640 medium (Nissui Pharmaceutical Co., Ltd., Tokyo, Japan) containing 10% fetal bovine serum (FBS) and antibiotics at 37 °C in a humidified atmosphere with 5% CO_2_. In vitro protein expression was detected as described previously at 1, 4, 10, and 22 h after incubation, with each complex including 1 µg of mRNA for 2 h in Opti-MEM without FBS [19].

### 2.5. Cytotoxicity of the Complexes

The B16 cells were cultured on a 96-well plate at a density of 3 × 10^3^ cells/well. The cells were incubated with various complexes containing 1 µg of mRNA for 2 h in Opti-MEM I medium. The lactate dehydrogenase (LDH) levels in the medium were then determined using a Cytotoxicity LDH Assay Kit (Dojindo Laboratories, Kumamoto, Japan) according to the manufacturer’s instructions. The percentages of the LDH levels compared with cells treated with the lysis buffer included in the kit were shown as the result.

### 2.6. Interaction of the Complexes with Erythrocytes

An agglutination study was performed as described previously [19]. The mRNA/PEI8 complex and mRNA/PEI8/γ-PGA12 complex were mixed with an erythrocyte suspension and agglutination was detected by microscopy (400× magnification).

### 2.7. Animals

Five-week-old male ddY mice were purchased from Japan SLC, Inc. (Shizuoka, Japan). All animal care and experimental procedures were performed according to the Guidelines for Animal Experimentation of Nagasaki University with approval from the Institutional Animal Care and Use Committee [approval number: 1710191419-4 (2017)].

### 2.8. In Vivo Protein Expression

The mice were administered with 400 µL of mRNA/PEI8/γ-PGA12 complex (containing 10, 20, 40, or 80 μg of mRNA) via intravenous injection. The mice were sacrificed, and the liver, kidney, spleen, heart, and lung were dissected at 3, 6, 12, and 24 h after the injection. Luciferase activities in the five organs (liver, kidney, spleen, heart, and lung) were measured according to the methods described in a previous report [19]. Luciferase activity was indicated as the relative light units (RLU) per gram of tissue.

Bioluminescence imaging was performed with a Xenogen IVIS Lumina System coupled with Living Image software for data acquisition (Xenogen Corp., Alameda, CA, USA). At 24 h after the injection of the mRNA/PEI8/γ-PGA12 complex, 12 mg of luciferin (Wako Pure Chemical Industries, Ltd., Osaka, Japan) dissolved in 300 μL PBS was administered by intraperitoneal injection. The luminescence from the live mice was then monitored.

### 2.9. Statistical Analysis

Statistical significance among the different groups was performed using Dunnett’s pairwise multiple comparison *t* test. *p* < 0.05 was considered to be statistically significant.

## 3. Results

### 3.1. Physicochemical Properties of the mRNA/PEI Complexes

The particle size and ζ-potential of the mRNA/PEI complexes are listed in Table 1. The mRNA/PEI complex with a charge ratio of 1:2 was aggregated, while the other mRNA/PEI complexes showed particle sizes of 39–85 nm. The addition of PEI increased the ζ-potentials of the mRNA/PEI complexes, reaching a plateau of around 50 mV at a ratio > 1:8.

**Figure 1:**
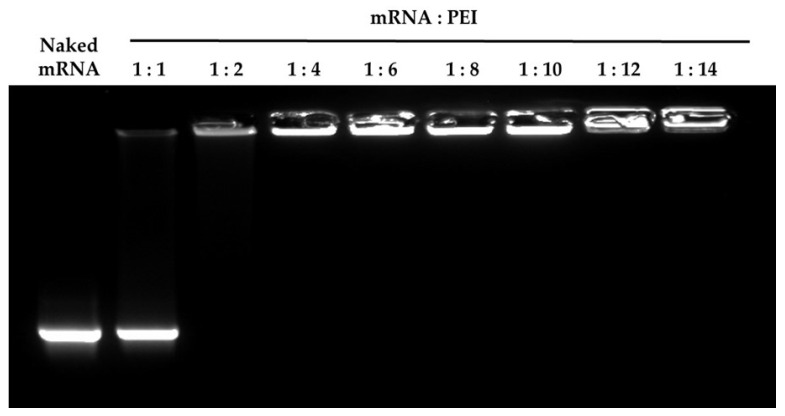
Electrophoretic analysis of the mRNA/PEI complexes. Each complex was applied to an agarose gel and subjected to electrophoresis. Retardation of the mRNA was visualized using GelRed.

Figure 1 illustrates the complex formation with a gel retardation assay. Naked mRNA was observed as a band on the agarose gel. In contrast, the complexed mRNA did not migrate out of the wells because none of the lanes containing the mRNA/PEI complexes produced any mRNA bands. This indicated that all of the mRNA/PEI complexes stably incorporated the mRNA at a ratio > 1:4.

### 3.2. In Vitro Protein Expression of the mRNA/PEI Complexes

**Figure 2:**
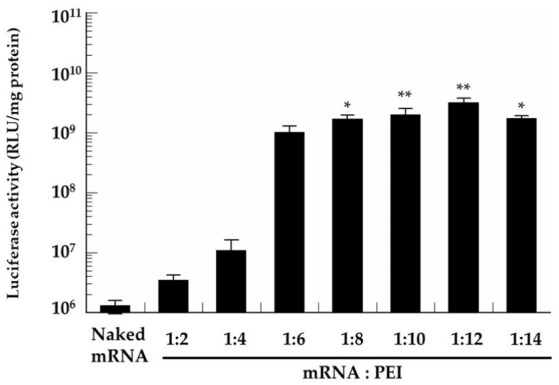
In vitro protein expression of the mRNA/PEI complexes at various charge ratios (1:2, 1:4, 1:6, 1:8, 1:10, 1:12, and 1:14) in B16 cells. B16 cells were incubated for 2 h with each complex. In vitro protein expression of the complexes was determined in the absence of fetal bovine serum. At 24 h following the addition of the complexes, luciferase activity in the cells was evaluated. Data represent the mean ± S.E. (*n* = 3). * *p* < 0.05, ** *p* < 0.01 vs. naked mRNA.

In this study, we used B16 cells, which have been widely used in various studies [20,21]. Figure 2 shows the luciferase activities of B16 cells treated with various mRNA/PEI complexes. Little luciferase activity was observed in the cells treated with naked mRNA. Luciferase activity increased with increasing PEI in a dose-dependent manner, reaching a plateau at a charge ratio of 1:8.

### 3.3. Physicochemical Properties of the mRNA/PEI8/γ-PGA Complexes

In this study, we selected the mRNA/PEI8 complex to prepare the mRNA/PEI/γ-PGA complex based on its physicochemical properties and efficiency of in vitro protein expression. To prepare the mRNA/PEI/γ-PGA complexes, γ-PGA was mixed with the mRNA/PEI8 complex at various charge ratios. The resulting particle sizes and ζ-potentials of these mRNA/PEI8/γ-PGA complexes are shown in Table 2. The mRNA/PEI8/γ-PGA4 complex was aggregated, while the other mRNA/PEI8/γ-PGA complexes had a particle size range of 44–81 nm. The ζ-potential decreased with an increasing ratio of γ-PGA to the mRNA/PEI8 complex, reaching a plateau at charge ratios > 1:8:10.

**Figure 3:**
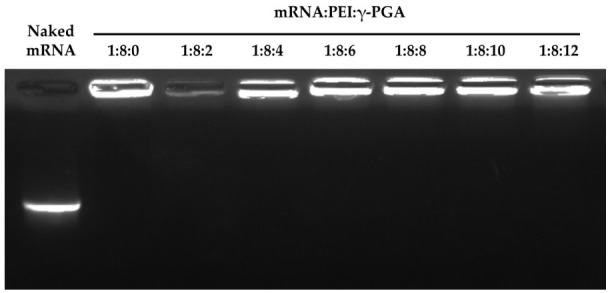
Electrophoretic analysis of the mRNA/PEI8/γ-PGA complexes. The complexes were applied to an agarose gel and subjected to electrophoresis. Retardation of the mRNA was visualized using GelRed.

The gel retardation assay was employed to examine whether the addition of γ-PGA dissociated the mRNA/PEI8 complex (Figure 3). No dissociated mRNA was observed for any of the complexes, indicating that the mRNA was stably contained within the complexes.

### 3.4. Cytotoxicity of the Complexes

**Figure 4:**
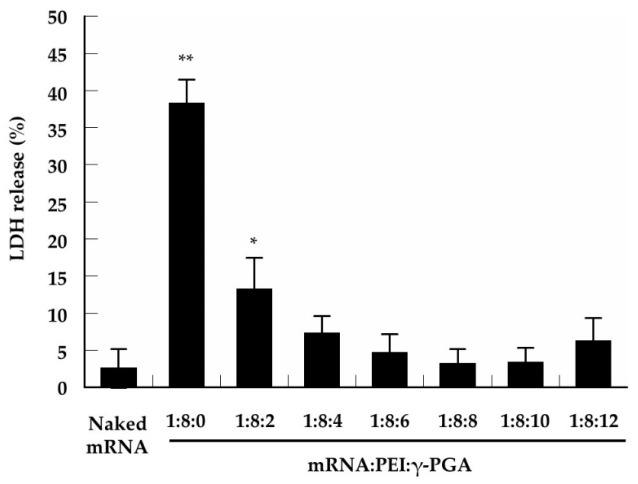
Cytotoxicity of the mRNA/PEI8 complex and the mRNA/PEI8/γ-PGA complexes at various charge ratios (1:8:2, 1:8:4, 1:8:6, 1:8:8, 1:8:10, and 1:8:12). Cytotoxicity was based on lactate dehydrogenase (LDH) release from B16 cells treated with each complex. Cells were incubated with the complexes for 2 h before the measurement of LDH in the culture medium. Data represent the mean ± S.E. (*n* = 8). * *p* < 0.05, ** *p* < 0.01 vs. naked mRNA.

Cytotoxicity was determined by adding the various complexes to B16 cells and measuring the release of LDH (Figure 4). Negligible LDH release was observed with the naked mRNA. The mRNA/PEI8 complex showed the strongest cytotoxicity compared with the naked mRNA (*p* < 0.01). The addition of γ-PGA to the mRNA/PEI8 complex markedly decreased the release of LDH (*p* < 0.05). There were no significant differences between the cytotoxicity of naked mRNA and that of the mRNA/PEI8/γ-PGA complexes with charge ratios over 1:8:4.

### 3.5. In Vitro Protein Expression of the mRNA/PEI8/γ-PGA Complexes

**Figure 5:**
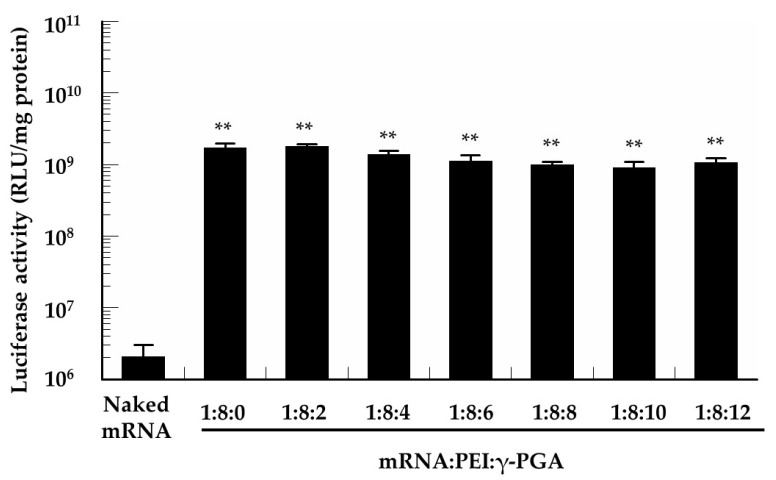
In vitro protein expression efficiency of the mRNA/PEI8/γ-PGA complexes at various charge ratios (1:8:0, 1:8:2, 1:8:4, 1:8:6, 1:8:8, 1:8:10, and 1:8:12). B16 cells were treated with each complex containing 1 µg of the mRNA for 24 h, following which, the luciferase activity was evaluated. Data represent the mean ± S.E. (*n* = 3). ** *p* < 0.01 vs. naked mRNA.

The B16 cells were treated with various mRNA/PEI8/γ-PGA complexes to determine their in vitro protein expression efficiency (Figure 5). All of the mRNA/PEI8/γ-PGA complexes showed high luciferase activity comparable to that of the mRNA/PEI8 complex regardless of their anionic surface.

**Figure 6:**
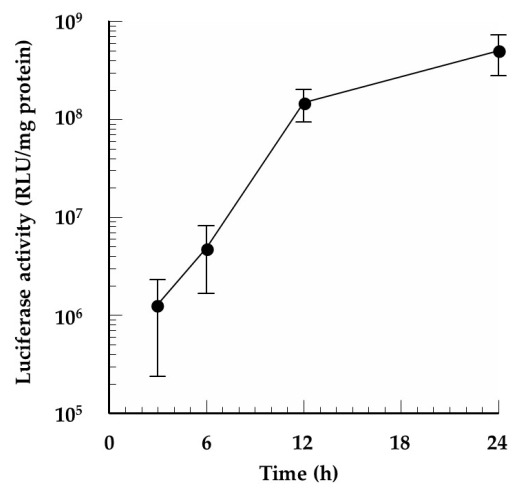
In vitro profile of the protein expression efficiency of mRNA/PEI8/γ-PGA12 in B16 cells. At 3, 6, 12, and 24 h following the addition of the complex, luciferase activity was evaluated. Data represent the mean ± S.E. (*n* = 3).

We selected the mRNA/PEI8/γ-PGA12 complex for further study based on its low cytotoxicity and high in vitro protein expression efficiency. Luciferase activity of the B16 cells was determined at different intervals over a 24 h period after the addition of the mRNA/PEI8/γ-PGA12 complex. Figure 6 shows that luciferase protein expression gradually increased over the 24-h period.

### 3.6. RNase Tolerance of the mRNA/PEI8 Complex and the mRNA/PEI8/γ-PGA12 Complex

**Figure 7:**
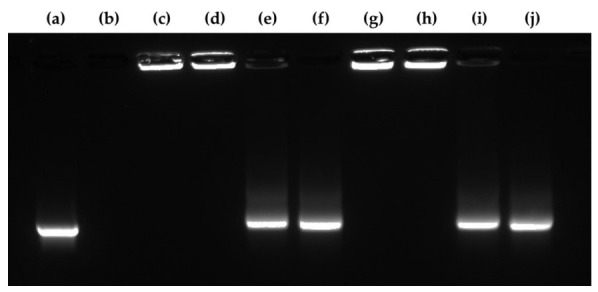
Electrophoretic analysis of naked mRNA and the mRNA/PEI8/γ-PGA12 complex following RNase treatment. The mRNA was visualized using GelRed. Heparin sulfate was added to the samples to evaluate the effect of complex dissociation. (**a**) Naked mRNA, (**b**) naked mRNA incubated with RNase, (**c**) mRNA/PEI8 complex, (**d**) mRNA/PEI8 complex incubated with RNase, (**e**) mRNA/PEI8 complex to which heparin was added, (**f**) mRNA/PEI8 complex incubated with RNase and to which heparin was added, (**g**) mRNA/PEI8/γ-PGA12 complex, (**h**) mRNA/PEI8/γ-PGA12 complex incubated with RNase, (**i**) mRNA/PEI8/γ-PGA12 complex to which heparin was added, (**j**) mRNA/PEI8γ-PGA12 complex incubated with RNase and to which heparin was added.

The mRNA/PEI8 complex, the mRNA/PEI8/γ-PGA12 complex, and naked mRNA were incubated with RNase and the stability of the mRNA was evaluated by agarose gel electrophoresis (Figure 7). The naked mRNA was completely degraded in the presence of RNase. No bands or fragments of mRNA were detected for the mRNA/PEI8 complex or the mRNA/PEI8/γ-PGA12 complex, either with or without RNase treatment. After complex dissociation by heparin sulfate, bands equivalent to the naked mRNA appeared for each complex both with and without RNase treatment.

### 3.7. Agglutination Study of the mRNA/PEI8 Complex and mRNA/PEI8/γ-PGA12 Complex

**Figure 8:**
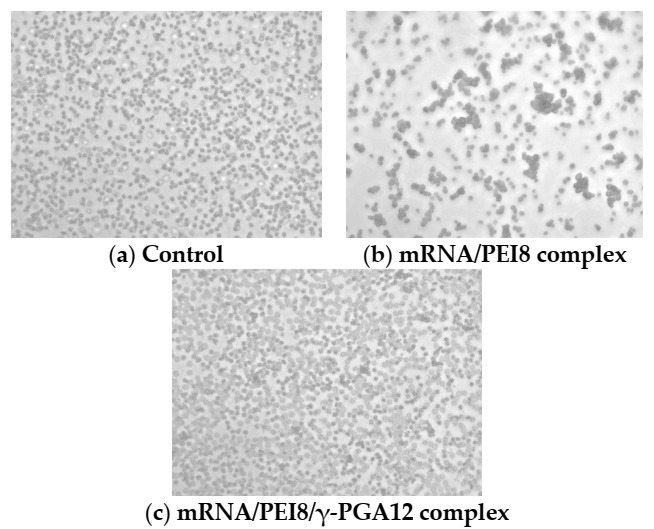
Agglutination study of phosphate-buffered saline (**a**), mRNA/PEI8 (**b**), and mRNA/PEI8/γ-PGA12 complex (**c**) with erythrocytes. After the complexes were added to the erythrocytes, agglutination was observed by phase-contrast microscopy (400× magnification).

The agglutination activity of the mRNA/PEI8/γ-PGA12 complex was determined with erythrocytes and observed by microscopy (400× magnification), as shown in Figure 8. The cationic complex of mRNA/PEI8 was agglutinated with the erythrocytes, which have an anionic membrane surface. In contrast, the anionic complex of mRNA/PEI8/γ-PGA12 showed little agglutination of the erythrocytes.

### 3.8. In Vivo Protein Expression of the mRNA/PEI8/γ-PGA12 Complex

**Figure 9:**
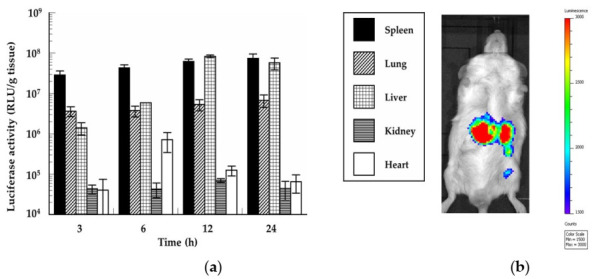
Protein expression of the mRNA/PEI8/γ-PGA12 complex containing 40 µg mRNA in mice after intravenous administration. (**a**) Luciferase activities in the liver, kidney, spleen, heart, and lung were determined by chemiluminescence. Data represent the mean ± S.E. (*n* = 4–8). (**b**) Light emission to the outside of the body was measured using an in vivo imaging system at 24 h after mRNA/PEI8/γ-PGA12 complex administration.

The in vivo protein expression of the mRNA/PEI8/γ-PGA12 complex was examined in mice. The complex for in vivo experiments, which was prepared in the 5% glucose solution, showed same size and ζ-potential as one for the in vitro experiments. The in vivo experiment of mRNA/PEI8 complex was not carried out because of its agglutination with erythrocytes. The luciferase activities in the five mouse organs were determined by chemiluminescence at 3, 6, 12, and 24 h after the intravenous administration of the mRNA/PEI8/γ-PGA12 complex (Figure 9a). The mRNA/PEI8/γ-PGA12 complex showed high luciferase activities in the liver and spleen and slight activity in the lung. This activity was maintained over the 24 h of observation. In the liver, the luciferase activity showed a marked increase by 12 h and this increase was maintained for the remainder of the 24 h. There was little luciferase activity in the heart or kidney. Strong light emission was observed from the liver and spleen in live mice using IVIS at 24 h after mRNA/PEI8/γ-PGA12 complex administration (Figure 9b).

**Figure 10:**
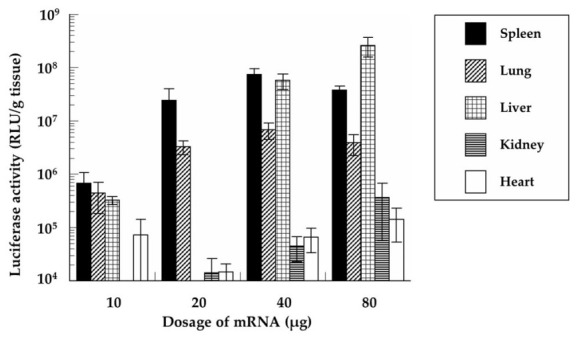
Protein expression of the mRNA/PEI8/γ-PGA12 complex in mice after the intravenous administration of various doses of mRNA. At 24 h following administration, the luciferase activities of the liver, kidney, spleen, heart, and lung were determined by chemiluminescence. Data represent the mean ± S.E. (*n* = 4–8).

Using a similar approach, the luciferase activities of the five organs were determined at 24 h after the intravenous administration of various doses of the mRNA/PEI8/γ-PGA12 complex (Figure 10). The low dose (10 µg mRNA) showed little protein expression in any of the tissues. Comparably high luciferase activities in the spleen were observed for doses of 20, 40, and 80 µg mRNA. The highest activity in the lung was observed at the dose of 40 µg mRNA. Furthermore, the luciferase activity in the liver increased with increasing doses of mRNA. There was little luciferase activity in the kidney or heart even in the presence of high doses.

## 4. Discussion

Gene delivery systems include viral vectors and non-viral vectors. Non-viral vectors have many advantages such as low cytotoxicity, low immunogenicity, no size limit, low cost, and reproducibility [22]. Among the various non-viral strategies that have been developed, cationic polymers are regarded as the most promising strategy because of their unique ability to electrostatically bind to genetic material and protect it from various enzymes [23].

PEI, which is a cationic polymer with repeating units composed of an amine group and an aliphatic spacer, has been widely investigated for gene delivery [24,25,26]. The ethyleneimine repeating units condense with genetic material, allowing the construction of nanoparticles that enhance gene delivery. In 1995, the first report of the successful use of this polymer for in vivo applications described the delivery of DNA into the brains of newborn mice [27]. The cationic complexes of pDNA with PEI were found to bind to receptors followed by gradual electrostatic zippering of the plasma membrane around the particle; this was sustained by the lateral diffusion of many syndecan molecules clustering into cholesterol-rich rafts [28]. In the endosomal compartment, PEI causes osmotic swelling because of the proton sponge effect, which enables efficient endosomal escape, protection from lysosomal degradation, and enhancement of protein expression [29].

In the present study, PEI condensed the mRNA to form complexes at various charge ratios, and the strong compaction of mRNA by PEI likely protected it from degradation by RNase. The mRNA/PEI2 complex, however, was aggregated. It is known that particles with a strong charge repel each other, while particles with a charge near neutrality have a weak repulsive force and bonding between these particles is considered to occur as a result of hydrophobic interactions. Furthermore, in this study, we used a theoretical charge ratio based on the phosphate groups of the mRNA, nitrogen groups of PEI, and carboxylate groups of γ-PGA. In practice, the ζ-potential of the mRNA/PEI1 complex had a negative charge, and the mRNA/PEI complexes presented a cationic surface at a ratio > 1:4. It appears that the cationic surface of the mRNA/PEI complexes became easily attached to the B16 cells, promoting cellular uptake, and resulting in protein expression largely via the proton sponge effect. In fact, the various mRNA/PEI complexes showed high luciferase activity in the B16 cells. Protein expression was increased by the addition of PEI in a dose-dependent manner, reaching a plateau at a charge ratio of more than 1:6.

The pDNA/PEI complex, however, has been reported to exhibit strong cytotoxicity and apoptosis induction. The pDNA/PEI complex is associated with marked LDH release within 1 h of exposure followed by a time-dependent increase [30]. Similar to this rapid LDH release, phosphatidylserine (PS) is redistributed rapidly (within 30 min) from the inner plasma membrane to the outer cell surface [30]. The translocation of PS is a hallmark of apoptosis and is associated with early necrotic-like cell damage [31,32,33,34].

In the present study, the complex of mRNA with PEI alone also showed strong cytotoxicity. Significantly higher LDH release from the B16 cells was observed with the mRNA/PEI8 complex compared with the naked mRNA. Furthermore, the mRNA/PEI8 complex showed marked agglutination after incubation with erythrocytes. This agglutination was likely caused by electrostatic interactions between the cationic complexes and anionic erythrocytes. Therefore, neutralization of the cationic complex surface must be achieved by the addition of anionic polymers to reduce the cytotoxicity and agglutination of the complexes.

Indeed, the addition of an anionic polymer, γ-PGA, reduced the strong cytotoxicity of the mRNA/PEI8 complexes, and the mRNA/PEI8/γ-PGA12 complex showed little agglutination. An anionic surface was confirmed for most of the γ-PGA complexes, and this property would have reduced the interaction of these complexes with the anionic cellular membrane. Little cytotoxicity was observed for the mRNA/PEI8/γ-PGA12 complex. These results were probably attributable to the low levels of interaction between this complex and the cells and erythrocytes. Furthermore, through compaction, the mRNA/PEI8/γ-PGA12 complex strongly protected the mRNA from RNase degradation, while rapid degradation was observed for the naked mRNA. Although the strong anionic polymer, heparin sulfate, pushed out the intact mRNA from the mRNA/PEI8/γ-PGA12 complex, the addition of anionic γ-PGA, however, did not release mRNA from the complex at any of the ratios tested.

We previously reported the unique behavior of the anionic complex of pDNA, PEI, and γ-PGA [13]. This anionic complex showed high protein expression in B16-F10 cells comparable with that of the cationic complex of pDNA/PEI regardless of its anionic surface, which would have reduced its interaction with the cellular membrane. This unique complex of pDNA has been useful in the development of a melanoma vaccine and malaria vaccine [14,18]. In the present study, strong protein expression was also confirmed for the mRNA/PEI/γ-PGA complex regardless of its anionic surface. We observed high protein expression in B16 cells already at 3 h after the addition of the mRNA/PEI8/γ-PGA12 complex. This early protein expression indicates that nuclear entry is not a requirement for uptake-a factor that poses a significant barrier to pDNA delivery.

After intravenous administration of the mRNA/PEI8/γ-PGA12 complex to mice, luciferase activity was detected in various organs. Strong protein expression was observed in the liver and spleen with only slight protein expression in the lung. The protein expression in the spleen and lung appeared to be saturated at an early stage because the expression levels detected at 3 h were maintained over the 24-h analysis period. The protein expression in the liver was increased by 12 h and was maintained for the remainder of the 24 h. The liver is composed of various cells such as Kupffer cells and hepatic parenchymal cells, and it is considered that the efficiency of cellular uptake and protein expression differ depending on the cell type. The increase of protein expression at an early stage may be caused by the slow uptake and large capacity of hepatic parenchymal cells. Phagocytosis by Kupffer cells would involve rapid degradation of the mRNA [35]. The strong luminescence produced by luciferase expression from the mRNA/PEI8/γ-PGA12 complex in the spleen and liver was also confirmed in live mice with an in vivo imaging system.

To examine the dose-dependence of protein expression, luciferase activity was determined in various tissues at 24 h after the intravenous administration of various doses of the mRNA/PEI8/γ-PGA12 complex. The low dose (10 µg mRNA) showed little protein expression in any of the tissues, suggesting that degradation reduced the mRNA levels. With mRNA doses of 20, 40, and 80 µg, comparable protein expression levels were detected in the spleen and lung. The processes of endocytosis and protein expression in the spleen and lung may be saturated at doses over 20 µg mRNA. In contrast, the luciferase activity in the liver increased with increasing doses of mRNA. The liver is a large organ and must have the capacity to uptake large quantities of the complex. These results suggest that the present complex may be useful for liver enzyme replacement therapy and vaccination via the spleen. Further experiments such as in vivo toxicity will be necessary for clinical use.

## 5. Conclusions

In this study, we newly constructed the mRNA/PEI8/γ-PGA12 complex for the effective delivery of mRNA into cells. This complex showed high in vitro protein expression in B16 cells without cytotoxicity and little agglutination with erythrocytes. Particularly strong protein expression was observed in the spleen and liver. These results indicate the potential usefulness of this mRNA complex for future clinical use.

## Figures and Tables

**Table 1:** Particle sizes and ζ-potentials of the mRNA/PEI complexes.

mRNA:PEI	Particle Size (nm)	ζ-Potential (mV)
1:1	39.4 ± 17.1	−8.9 ± 0.6
1:2	Aggregated	−0.2 ± 0.2
1:4	84.9 ± 10.6	36.1 ± 0.5
1:6	40.7 ± 4.2	42.9 ± 0.3
1:8	46.3 ± 6.4	47.4 ± 1.4
1:10	38.9 ± 10.1	51.2 ± 1.4
1:12	42.9 ± 21.4	54.4 ± 0.1

Each data represents the mean ± S.D. (*n* = 3).

**Table 2:** Particle sizes and ζ-potentials of mRNA/PEI8/γ-PGA complexes.

mRNA:PEI:γ-PGA	Particle Size (nm)	ζ-Potential (mV)
1:8:0	46.3 ± 6.4	47.4 ± 1.4
1:8:2	45.1 ± 2.6	36.2 ± 0.3
1:8:4	Aggregated	17.0 ± 0.7
1:8:6	80.4 ± 8.0	−10.0 ± 0.1
1:8:8	44.7 ± 7.9	−27.2 ± 3.0
1:8:10	46.5 ± 12.4	−31 ± 0.3
1:8:12	52.0 ± 7.3	−35.5 ± 1.6

Each data represents the mean ± S.D. (*n* = 3).

## Data Availability

Not applicable.
